# miR‐204‐5p suppresses hepatocellular cancer proliferation by regulating homeoprotein SIX1 expression

**DOI:** 10.1002/2211-5463.12363

**Published:** 2018-01-15

**Authors:** Yi Chu, Mingzuo Jiang, Feng Du, Di Chen, Tao Ye, Bing Xu, Xiaowei Li, Weijie Wang, Zhaoyan Qiu, Haiming Liu, Yongzhan Nie, Jie Liang, Daiming Fan

**Affiliations:** ^1^ State Key Laboratory of Cancer Biology & Institute of Digestive Diseases Xijing Hospital The Fourth Military Medical University Xi'an China; ^2^ State Key Laboratory of Military Stomatology National Clinical Research Center for Oral Diseases Shannxi key Laboratory of Oral Diseases School of Stomatology The Fourth Military Medical University Xi'an China; ^3^ Department of General Surgery the General Hospital of the people's Liberation Army Beijing China; ^4^ College of Computer Science and Technology Symbolic Computation and Knowledge Engineering of Ministry of Education Jilin University Changchun China

**Keywords:** cell cycle, HCC proliferation, miR‐204‐5p, SIX1

## Abstract

Fewer than 30% of patients with hepatocellular carcinoma (HCC) are eligible to receive curative therapies, and so a better understanding of the molecular mechanisms of HCC is needed to identify potential therapeutic targets. The role of microRNA (miRNA) in modulating tumour progression has been demonstrated, and therapies targeting miRNA appear promising. miR‐204‐5p has been shown to function in numerous types of cancer, but its role in HCC remains unclear. In this study, we found that miR‐204‐5p expression was downregulated in cancerous HCC tissues compared to nontumour tissues. Kaplan–Meier survival curve analysis also showed that low expression of miR‐204‐5p predicted worse outcomes of HCC patients. In addition, miR‐204‐5p expression was significantly lower in HCC cell lines. The function of miR‐204‐5p was also assessed both *in vitro* and *in vivo*. We demonstrated that ectopic expression of miR‐204‐5p in HCC cell lines inhibited HCC cell proliferation and clonogenicity using CCK8, BrdU and colony‐forming assays, while the inhibition of miR‐204‐5p enhanced proliferation and clonogenicity. Further *in vivo* studies in mice further confirmed the proliferation capacity of miR‐204‐5p. We also identified sine oculis homeobox homologue 1 (*SIX1*) as a direct target of miR‐204‐5p and showed that it was inversely correlated with miR‐204‐5p in both human and mouse HCC tissues. Transfection of miR‐204‐5p mimics in BEL‐7404 cells blocked the cell cycle by inhibiting the expression of cyclin‐D1 and cyclin‐A1, cell cycle‐related factors regulated by SIX1. More importantly, overexpression of the 3′UTR mutant *SIX1* but not the wild‐type *SIX1* abolished the suppressive effect of miR‐204‐5p, and downregulated SIX1 in BEL‐7402 cells that transfected with miR‐204 inhibitors could partly block the inhibitory effect of miR‐204‐5p on proliferation. Thus, we have demonstrated that miR‐204‐5p suppresses HCC proliferation by directly regulating SIX1 and its downstream factors.

**Abbreviations**
BrdU: 5‐bromo‐2‐deoxyuridine
CCK8: Cell Counting Kit‐8
FACS: fluorescence‐activated cell sorting
HCC: hepatocellular carcinoma
LV: lentivirus
miRNA: microRNA
qRT‐PCR: quantitative real‐time PCR
siRNA: small interfering RNA
SIX1: sine oculis homeobox homologue 1
UTR: untranslated region

Hepatocellular carcinoma (HCC) is by far the most common primary liver cancer and is the second most common cause of cancer deaths in men and the fifth leading cause of cancer deaths in women 1. There are more than half a million new diagnoses of HCC every year, and multiple well‐documented aetiological factors contribute to the progression of HCC, including alcohol, aflatoxin, chronic infection with hepatitis B or C virus (HBV, HCV) and obesity due to fatty diets 2, 3. However, the underlying mechanisms of HCC remain unclear, and fewer than 30% of HCC patients are eligible to receive curative therapies 4. Therefore, it is imperative to elucidate the molecular mechanisms of HCC proliferation and identify potential therapeutic targets.

MicroRNA (miRNA) are a class of endogenous noncoding short RNA that exert regulatory functions by binding with the 3′ untranslated region (UTR) of complementary mRNA. Currently, there are over 2500 potential human microRNA recorded in miRBase 5. Many of them are involved in crucial biological processes, and an increasing number of microRNA have been identified as oncogenes or oncosuppressors by regulating relevant targets. Therefore, inhibition of specific microRNA could be an effective therapeutic approach for various cancers. miR‐204‐5p has been reported to be involved in tumour progression by regulating numerous oncogenic activities, including proliferation, metastasis, chemotherapy resistance and autophagy 6, 7, 8, 9, but its function in the HCC is still unclear.

Sine oculis homeobox homologue 1 (SIX1) is a member of the SIX family, which is highly conserved in many species 10. The *SIX1* gene, which is highly expressed throughout embryogenesis, encodes a homeodomain‐containing transcription factor that is essential for the development of human organs 11 but shows little expression in adult tissues 12. However, SIX1 was shown to be overexpressed in various types of cancer 13, 14, 15, 16 and is known to be involved in tumour progression by regulating c‐myc, cyclin‐D1 and cyclin‐A1 17, 18. To date, analysis of SIX1 has revealed that SIX1 is critical for metastasis by regulating EMT both *in vitro* and *in vivo*. In addition, SIX1 overexpression promotes tumour lymphangiogenesis by coordinating TGF‐β signals that increase expression of VEGF‐C. Notwithstanding these significant findings of SIX1, the mechanisms of SIX1 regulation of hepatocellular cancer proliferation are still largely unknown. miR‐204‐5p was reported to suppress tumour proliferation by regulating SIX1 in breast cancer and NSCLC 19, 20. However, whether miR‐204‐5p and SIX1 exert the same function in HCC has not been demonstrated.

In this study, we investigated the potential functions of miR‐204‐5p in HCC proliferation and the relationship between miR‐204‐5p and SIX1. We found that miR‐204‐5p was downregulated in HCC clinical samples and suppressed HCC proliferation by directly decreasing SIX1 expression. Our data provided the first evidence that miR‐204‐5p regulated HCC proliferation through the miR‐204‐5p/SIX1 pathway, suggesting that miR‐204‐5p and SIX1 could be potential therapeutic targets for HCC treatment.

## Results

### miR‐204‐5p is downregulated in human HCC tissues and cell lines

**Figure 1:**
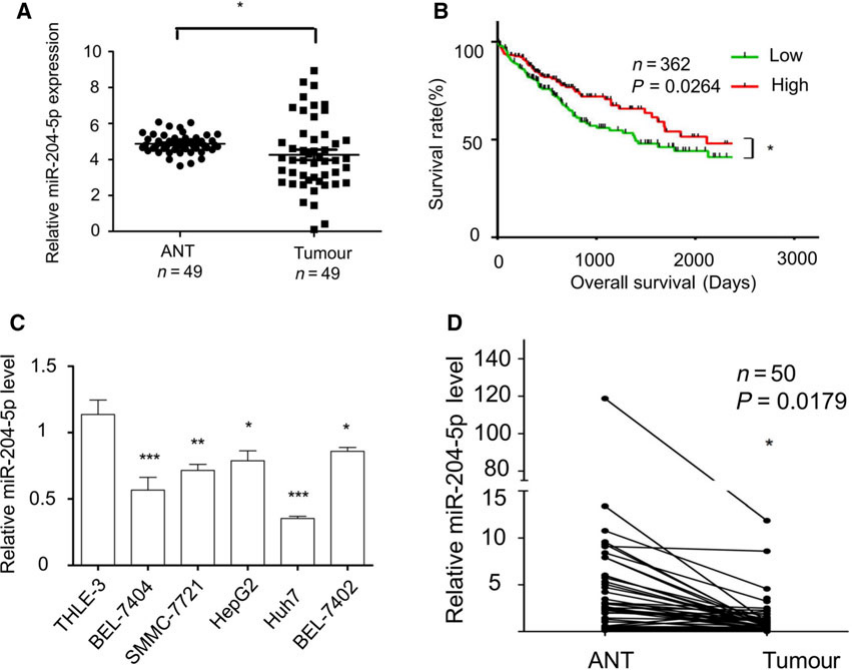
miR‐204‐5p was downregulated in HCC. (A) Expression of miR‐204‐5p based on TCGA database (*n* = 49). (B) Kaplan–Meier survival curves of miRNA in TCGA database (*n* = 362). (C) The qRT‐PCR analysis of miR‐204‐5p in five HCC cell lines (BEL‐7404, SMMC‐7721, HepG2, Huh7 and BEL‐7402) and one normal liver cell line (THLE‐3). (D) Comparison of miR‐204‐5p expression levels between HCC tissues and adjacent normal tissues in 50 HCC patients. Values represent mean ± SEM. *P < 0.05; **P < 0.01; ***P < 0.001.

Although miR‐204‐5p was shown to be decreased in many types of malignancy 19, 21, 22, the expression level of miR‐204‐5p in HCC tissues has not been reported. To investigate the expression level of miR‐204‐5p in HCC, we analysed the data from TCGA online data set (http://cancergenome.nih.gov/). The results showed that miR‐204‐5p was downregulated in HCC compared to nontumour tissues in TCGA online data set (Fig. 1A). In addition, we used Kaplan–Meier survival curves to analyse the 5‐year overall survival rates of patients with HCC and found that the 5‐year overall survival rates of patients with low miR‐204‐5p levels were significantly worse than those of HCC patients with relatively high miR‐204‐5p levels (Fig. 1B).

To further examine the expression level of miR‐204‐5p in HCC, we compared miR‐204‐5p levels in five HCC cell lines, namely BEL‐7404, SMMC‐7721, HepG2, Huh7 and BEL‐7402, and an immortalized human normal liver cell line, THLE‐3. miR‐204‐5p was significantly decreased in all five HCC cell lines compared with the normal cell line (Fig. 1C). The expression level of miR‐204‐5p was also strongly downregulated in 46 (92%) of 50 human HCC tissues compared with their normal counterparts (Fig. 1D).

All these data suggested that miR‐204‐5p was downregulated in human HCC tissues and HCC cell lines.

### miR‐204‐5p suppresses HCC cell proliferation *in vitro* and *in vivo*

**Figure 2:**
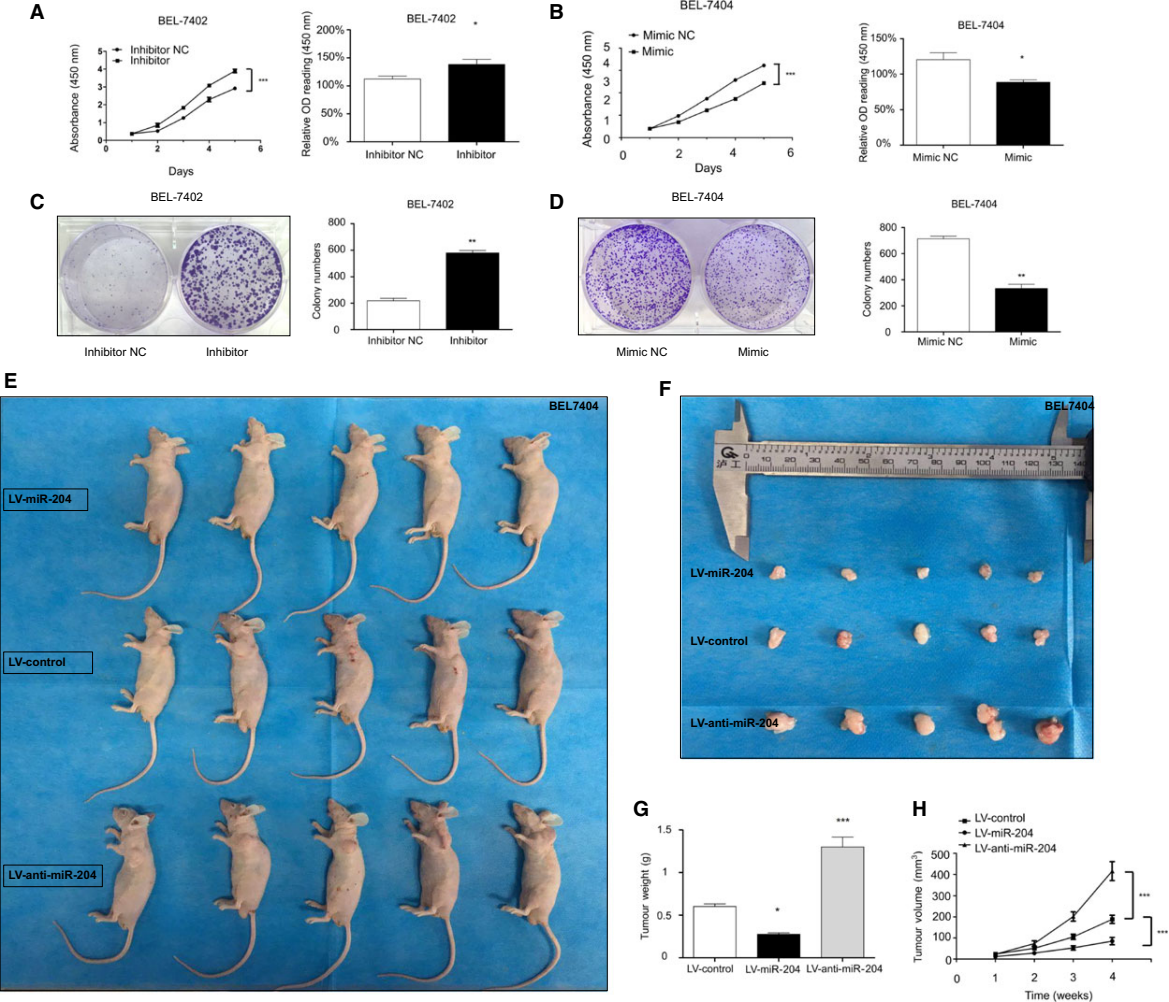
miR‐204‐5p suppressed HCC proliferation *in vitro* and *in vivo*. (A, B) CCK8 assay and BrdU assay of BEL‐7402(A)‐ and BEL‐7404(B)‐transfected cells. (C, D) Colony‐forming assay and semiquantitative data of BEL‐7402(C)‐ or BEL‐7404(D)‐transfected cells. (E, F) Representative images of tumours of BEL‐7404 cells transfected with miR‐204‐5p mimics and inhibitors. (G) Tumour weights were measured after removal. (H) Growth curve of tumour volumes in the xenograft model was measured every week. Values represent the mean ± SEM. *P < 0.05; **P < 0.01; ***P < 0.001.

To further investigate the function of miR‐204‐5p in HCC proliferation, we selected HCC cell lines with high and low levels of miR‐204‐5p, BEL‐7402 and BEL‐7404, respectively, for further study. To confirm the inhibitory effect of miR‐204‐5p on proliferation, we transfected a miR‐204‐5p inhibitor into BEL‐7402 cells, and cell growth was evaluated by CCK8 and BrdU assays. According to the manufacturer's instructions, the cells were incubated with CCK8 and BrdU detection antibody for 4 h. As expected, inhibition of miR‐204‐5p promoted HCC cell growth (Fig. 2A) and colony formation (Fig. 2C). miR‐204‐5p mimic or negative control (NC) was transfected into BEL‐7404 cells. The results showed that overexpression of miR‐204‐5p dramatically repressed cell growth (Fig. 2B). Colony‐forming assays also demonstrated that miR‐204‐5p mimics significantly decreased colony formation efficiency (Fig. 2D). Collectively, these data suggested that miR‐204‐5p plays an oncosuppressor role in HCC *in vitro*.

To determine whether miR‐204‐5p had the same oncosuppressor role *in vivo*, we subsequently examined its function using xenograft tumour models in nude mice. BEL‐7404 cells with stable overexpression of miR‐204‐5p(LV‐miR‐204), cells with downregulation of miR‐204‐5p(LV‐anti‐miR‐204) and negative control (LV‐control) cells were subcutaneously injected into the 6‐week‐old nude mice at a dose of 2 × 10^6^ mL^−1^. The tumours in the LV‐miR‐204 group grew more slowly than those in the LV‐control group (Fig. 2H). However, the tumours in mice injected with BEL‐7404 cells in which miR‐204‐5p was inhibited grew faster than those of mice injected with NC. After 4 weeks, the tumour sizes of nude mice were significantly decreased in the miR‐204‐5p overexpression group, while they were increased in the miR‐204‐5p downregulated group compared with the control group (Fig. 2E–H). These results further confirmed our conclusion that miR‐204‐5p suppressed tumour growth *in vivo*.

### miR‐204‐5p slows down HCC cell cycle progression

**Figure 3:**
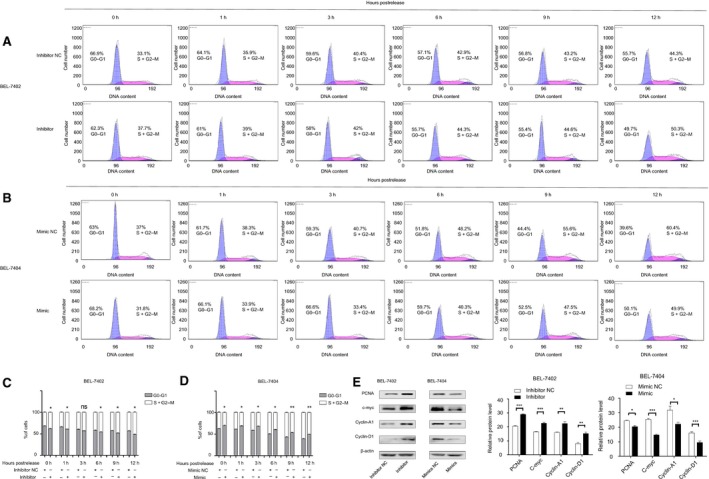
Flow cytometry of propidium iodide‐stained BEL‐7402(A) and BEL‐7404(B) cells demonstrates a slower progression through the cell cycle when miR‐204‐5p was upregulated. The numbers of G0‐G1 and S+G2 M represent percentage of cells in each phase. (C, D) Statistical analysis of the proliferation index of each cell line. (E) The classical proliferation downstream factor protein levels and semiquantitative data of BEL7402 and BEL7404 cells after transfection were examined by western blot. Values represent the mean ± SEM. *P < 0.05; **P < 0.01; ***P < 0.001.

Considering the significance of the cell cycle in the regulation of tumour proliferation, we investigated the antiproliferative effect of miR‐204‐5p further. To determine whether miR‐204‐5p could contribute to cell cycle functions, we used flow cytometry to analyse the cell cycle of HCC cells undergoing different treatments. We used the experimental strategy of cell synchronization to perform the cell cycle study. Elimination of serum from the culture medium for approximately 24 h resulted in the accumulation of cells at G1 phase 23. Cells were incubated with serum‐free Dulbecco's modified Eagle's medium (DMEM) for 24 h for cell synchronization. Then, the cells were treated with 10% FBS‐containing DMEM, trypsinized and washed at 0, 1, 3, 6, 9 and 12 h postrelease from FBS‐free DMEM. Next, the cells were stained with propidium iodide solution and incubated at 37 °C in the dark for 30 min for flow cytometry analysis. The cells were only treated with the mimic or inhibitor transfection so the sub‐G1 peak was not detectable. According to the technicians' advices, we have gated out the sub‐G1 population because it had negligible effects on the final results. The scale on the *y*‐axis was kept constant throughout for better visualization of our results. As shown in Fig. 3, from the fluorescence‐activated cell sorting (FACS) histograms, we observed that the cell numbers in G2 + M phase increased after release from the starving state. We also found that downregulation of miR‐204‐5p in BEL‐7402 cell lines enhanced cell cycle progression and resulted in an increase in the proportion of cells in the S‐G2/M phase (Fig. 3A), whereas overexpression of miR‐204‐5p in the BEL‐7404 cell lines resulted in a significant decrease in the proportion of S‐G2/M cells and slowed the cell cycle progression (Fig. 3B). Numbers of G0‐G1 and S+G2/M cells in Fig. 3 represent the percentage of cells in each phase, and these numbers could be used as a proliferative index of each cell line for further statistical analysis (Fig. 3C,D). We next assessed classical tumour proliferation proteins, including PCNA, c‐myc, cyclin‐A1 and cyclin‐D1, by western blot. Western blot analysis showed that upregulation of miR‐204‐5p decreased PCNA, c‐myc, cyclin‐A1 and cyclin‐D1 levels, and their expression levels were increased when miR‐204‐5p was inhibited (Fig. 3E). Taken together, the cell cycle distribution analysis shows that, in agreement with the expression of cyclin‐D1 and cyclin‐A1, miR‐204‐5p significantly slowed down cell cycle progression in HCC cells.

### miR‐204‐5p targets the 3′‐UTR of the *SIX1* homeobox gene

We demonstrated that miR‐204‐5p plays an important role in HCC proliferation. To further identify the mechanism of miR‐204‐5p in HCC, we used miRanda bioinformatics analysis to find possible targets of miR‐204‐5p. Among the top 50 targets predicted by miRanda, SIX1, a transcription factor reported to be involved in the proliferation and metastasis of several cancers, stood out among the numerous candidates. *SIX1* harbours a conserved miR‐204‐5p site in its 3′UTR and has been confirmed to be regulated by miR‐204‐5p in breast cancer and NSCLC 19, 20. Moreover, it has been reported that SIX1 overexpression results in an acceleration in cell cycle progression that occurs as early as the G1/S transition by reactivation of cyclin‐A1 and cyclin‐D1 18, 24. However, this mechanism has not been demonstrated in HCC.

**Figure 4:**
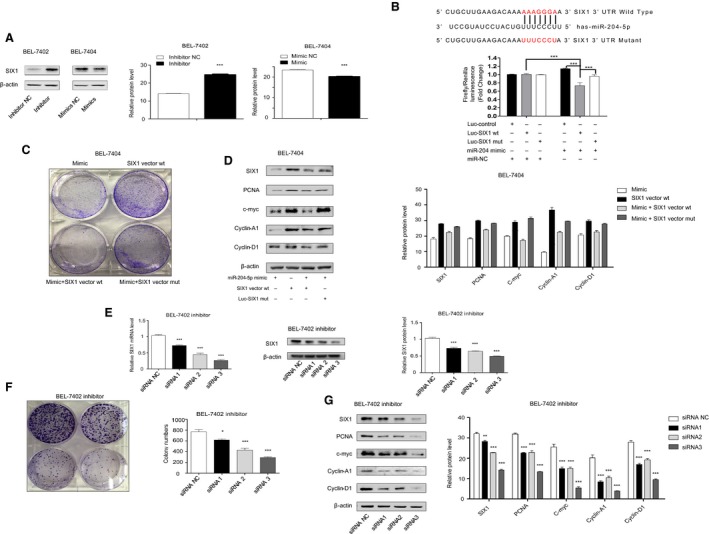
miR‐204‐5p directly targeted *SIX1* in HCC cells. (A) SIX1 protein levels and semiquantitative data of BEL7402 and BEL7404 cells after transfection were examined by western blot. (B) Dual‐luciferase assay. BEL7402 cells were cotransfected with hsa‐miR‐204‐5p or miR‐NC and *SIX1* plasmid with wild‐type or mutant 3′UTR. Firefly luciferase activity was normalized to *Renilla* luciferase activity. (C) Gain‐of‐function assays were performed using miR‐204‐5p mimics, *SIX1* overexpression vector alone, miR‐204‐5p mimics combined with *SIX1* overexpression vector and miR‐204‐5p mimics combined with 3′ UTR mutant SIX1 overexpression vector. (D) The classical proliferation downstream factor protein levels and semiquantitative data of BEL7404 cells after gain‐of‐function treatment were examined by western blot. (E) The efficiency of *SIX1* siRNA was examined by qRT‐PCR and western blot. (F) Rescue assay. The colony‐forming assay and semiquantitative analysis were performed after BEL‐7402 cells transfected with miR‐204‐5p inhibitors were treated with *SIX1* siRNA. (G) The classical proliferation downstream factor protein levels and semiquantitative data of BEL7402 inhibitor cells after transfection with *SIX1* siRNA were examined by western blot. Values represent the mean ± SEM. *P < 0.05; **P < 0.01; ***P < 0.001.

We examined SIX1 protein expression levels in two transfected cell lines by western blot analysis. SIX1 protein expression significantly decreased in cells that overexpressed miR‐204‐5p and increased in cells with miR‐204‐5p inhibition (Fig. 4A).

To further determine whether miR‐204‐5p could directly bind to the 3′UTR of *SIX1* mRNA, a dual‐luciferase reporter assay was employed. The results suggested that miR‐204‐5p could suppress reporter gene activity of the wild‐type 3′UTR but not the mutant (MUT) type (Fig. 4B), which indicated that *SIX1* was a direct target of miR‐204‐5p in HCC cells.

Gain‐of‐function assays were performed using miR‐204‐5p mimics, SIX1‐overexpressing vector alone, miR‐204‐5p mimics combined with the *SIX1*‐overexpressing vector and miR‐204‐5p mimics combined with the 3′UTR MUT *SIX1*‐overexpressing vector. The results of colony‐forming assays showed that the miR‐204‐5p could suppress cell proliferation and clonogenicity (Fig. 4C). More importantly, the 3′UTR MUT *SIX1*‐overexpressing vector but not the wild‐type *SIX1*‐overexpressing vector abolished the suppressive effect of miR‐204‐5p. We also examined the downstream factors after *SIX1* was overexpressed and found that only the MUT‐type *SIX1* overexpression group showed increased protein levels of PCNA, c‐myc, cyclin‐A1 and cyclin‐D1 (Fig. 4D).

Furthermore, we treated BEL‐7402 cells transfected with the miR‐204‐5p inhibitor with *SIX1* siRNA. The siRNA efficiencies of three siRNA were qualified by qPCR and western blot (Fig. 4E). Importantly, cells treated with *SIX1* siRNA reversed the effects of anti‐miR‐204‐5p on BEL7402 cells (Fig. 4F). We also examined the downstream factors after *SIX1* silencing and found that the protein levels of PCNA, c‐myc, cyclin‐A1 and cyclin‐D1 were also recovered (Fig. 4G). The results of loss‐of‐function assays were consistent with the gain‐of‐function assays and showed that the suppressive effect of miR‐204‐5p on HCC cell proliferation and clonogenicity is dependent on SIX1 and its downstream elements.

Thus, we demonstrated that miR‐204‐5p suppresses hepatocellular cancer proliferation by targeting the 3′UTR of the *SIX1* homeobox gene.

### Validation of the relationship between miR‐204‐5p and SIX1 in human samples and mouse model

**Figure 5:**
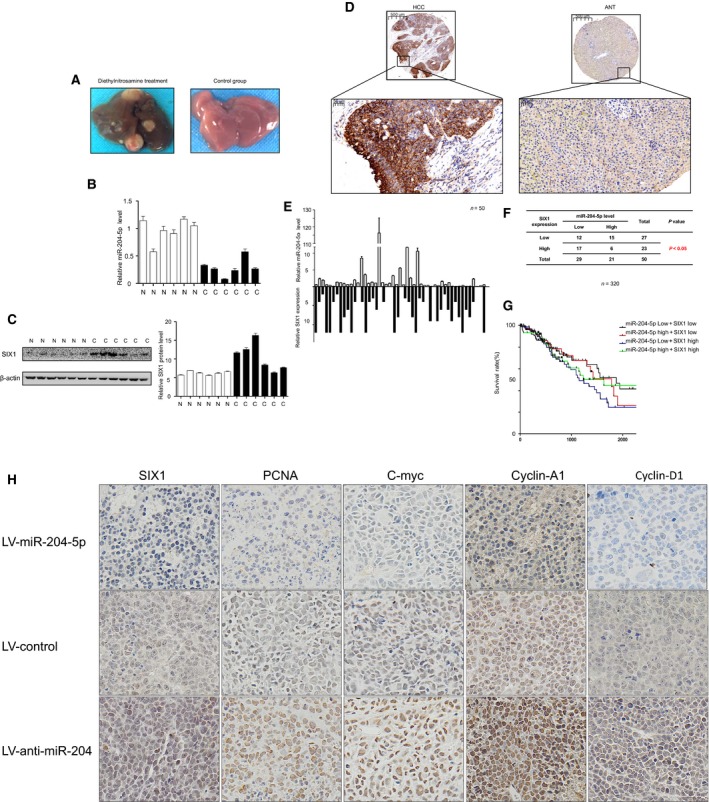
Validation of the relationship between miR‐204‐5p and SIX1 in human samples and mouse model. (A) Representative images of the diethylnitrosamine‐induced mouse HCC model. (B) Relative miR‐204‐5p expression levels in diethylnitrosamine‐induced mouse HCC model. (C) The SIX1 protein levels and semiquantitative data of diethylnitrosamine‐induced mouse HCC model. (D) Representative IHC staining of SIX1 in 50 HCC and adjacent normal liver samples. (E, F) Correlation between miR‐204‐5p level and SIX1 expression in 50 HCC patients. (G) Correlation of the combination of miR‐204‐5p and SIX1 expression with overall survival. (H) Representative images of IHC analysis of the tumour tissue sections in mouse xenograft to evaluate the levels of SIX1 and other downstream factors. Values represent the mean ± SEM.

To explore whether the mechanism also functions *in vivo,* we further used a diethylnitrosamine (DEN)‐induced mouse HCC model to confirm our hypothesis. The 14‐day‐old mice were intraperitoneally injected with DEN and sacrificed at 36 weeks (Fig. 5A). We found that miR‐204‐5p was largely decreased in the DEN group liver samples compared with the control group (Fig. 5B). We also evaluated SIX1 expression level, and the results showed that SIX1 protein levels were increased in DEN group liver samples (Fig. 5C), which showed an opposite trend compared with miR‐204‐5p.

We finally evaluated SIX1 expression in 50 human HCC samples to validate the relationship between miR‐204‐5p and SIX1 using immunohistochemistry (IHC). Remarkably, SIX1 was upregulated in HCC samples compared with adjacent normal tissues (Fig. 5D) and was inversely correlated with miR‐204‐5p expression level (Fig. 5E,F).

Staining analysis revealed a negative correlation between miR‐204‐5p and SIX1 expressions; thus, we investigated whether the prediction of HCC prognosis was more accurate using the combined expression of miR‐204‐5p and SIX1. We analysed the overall survival rate using the information of all‐cause mortality in TCGA database. Patients were subsequently divided into four groups: group 1, low expression of miR‐204‐5p and high expression of SIX1; group 2, high expression of miR‐204‐5p and low expression of SIX1; group 3, high expression of miR‐204‐5p and high expression of SIX1; and group 4, low expression of miR‐204‐5p and low expression of SIX1. As expected, the results showed that HCC patients with low levels of miR‐204‐5p and high levels of SIX1 had the poorest prognosis (Fig. 5G).

We also performed IHC on the tumour tissue sections in mouse xenografts to evaluate the levels of SIX1 and other downstream factors. The results showed that SIX1 and other downstream factors showed the highest expression level in the LV‐anti‐miR‐204 group and the lowest expression level in the LV‐miR‐204 group (Fig. 5H), which further validated our hypothesis.

These data further confirmed that miR‐204‐5p could regulate SIX1 in HCC progression.

## Discussion

Because of the unclear underlying mechanism, HCC is often diagnosed in the late stage, which prevents the use of standard treatments such as surgical resection and liver transplantation. Hence, it is necessary to elucidate the mechanism of HCC progression and develop potential new therapies.

MicroRNA have been reported to be associated with numerous human neoplasms, and targeting oncogenic microRNA is a possible treatment approach 25. miRNA mimics and inhibitors could be used as a new class of antitumour treatment 26. Various microRNA were shown to act as tumour suppressors or oncogenes in HCC, including miR‐21, miR‐101 and miR‐139 27. Yin *et al*. 21 reported that miR‐204‐5p acts as a tumour suppressor in colorectal cancer, which prompted us to investigate the potential role of miR‐204‐5p in HCC. In the present study, we found that the levels of a specific microRNA, miR‐204‐5p, were largely decreased in HCC tissues through RT‐qPCR.

Luo *et al*. 28 recently reported that miR‐204‐5p was associated with clinicopathological features of HCC; however, the direct targets and regulatory factors of miR‐204‐5p require further investigation. Additionally, Jiang *et al*. provided an overview of miR‐204‐5p in the malignant behaviours of HCC. They confirmed that miR‐204‐5p could regulate HCC malignancy by targeting SIRT1 29. However, we believe the targets of miR‐204‐5p may not be unique. To further study the function of miR‐204‐5p in HCC proliferation, we performed a series of experiments *in vitro*. The results showed that overexpression of miR‐204‐5p suppressed the cell proliferation, while the inhibition of miR‐204‐5p promoted HCC cell proliferation. We also confirmed this hypothesis in a third cell line, Huh7, and found that the antiproliferative function of miR‐204‐5p is widespread in HCC cells (Fig. S1). Moreover, a subcutaneous xenograft mouse model demonstrated that miR‐204‐5p could suppress HCC proliferation *in vivo*. Therefore, our data suggested that miR‐204‐5p is an antitumour factor in HCC.

To better understand the mechanism of miR‐204‐5p in HCC, we used an online microRNA database (miRanda) and identified *SIX1* as its downstream target gene. SIX1, which has been reported to be involved in various cancer progressions and correlated with poor prognosis in breast cancer 30, acts as a tumour‐promoting factor by regulating c‐myc, cyclin‐D1 and cyclin‐A1. In our study, we first demonstrated that SIX1 was upregulated in HCC samples and was inversely correlated with miR‐204‐5p expression level. MicroRNA regulate target mRNA by directly binding to the 3′UTR.

Therefore, we next performed dual‐luciferase reporter assays to determine whether miR‐204‐5p can directly target *SIX1* in HCC. The results suggested that miR‐204‐5p could suppress reporter gene activity of wild‐type 3′UTR but not MUT type, which indicates that miR‐204‐5p can directly target the 3′UTR of the *SIX1* homeobox gene. The downstream factors regulated by SIX1 showed the same change as SIX1 using western blot. Therefore, we concluded that miR‐204‐5p directly regulates SIX1 and its downstream factors.

Finally, we validated the relationship between miR‐204‐5p and SIX1 in human samples and mouse models. The results showed that miR‐204‐5p was decreased in both human and mouse HCC samples and showed an opposite tendency compared with SIX1.

Using TCGA database, we found the HCC patients with lower level of miR‐204‐5p and higher level of SIX1 had the poorest prognosis. However, we also found the patients with high level of miR‐204‐5p and low level of SIX1 also had the poorest prognosis. Given that in the late stage of HCC, the prognosis could be affected by various factors, such as age, physical and mental state, metastasis and complications. We believe that in the late stage of HCC, the prognosis could be too difficult to predict using just one pair of factors. We also showed in Fig. 5G that the patients with lower levels of miR‐204‐5p and higher levels of SIX1 had much poorer prognosis than the other three groups before 1800 days, approximately 5 years, which is a commonly used prognostic clinical time‐point. In addition, the patients with lower levels of miR‐204‐5p and higher levels of SIX1 always had the poorest prognosis. Based on the cut‐off point of 1800 days, we concluded that HCC patients with lower levels of miR‐204‐5p and higher levels of SIX1 had the poorest prognosis.

As it is known that several established aetiological factors could contribute to the progression of HCC, including alcohol or aflatoxin, chronic infection with HBV, HCV or obesity due to fatty diets, the accurate function of miR‐204‐5p in different subgroups of HCC needs to be further investigated.

Notably, we found that BEL‐7402 transfected with the miR‐204‐5p inhibitor and *SIX1* siRNA partially reversed the effects of anti‐miR‐204‐5p, which suggests that miR‐204‐5p might regulate other pathways to exert its functions in HCC proliferation and requires further investigation.

In summary, the results of our study identified a new mechanism in which miR‐204‐5p suppressed HCC proliferation by directly regulating SIX1 and its downstream factors both *in vitro* and *in vivo*, suggesting that these molecules may be potential targets for HCC treatment.

## Materials and methods

### Bioinformatics analysis

miRNA expression and 5‐year survival profiling data sets were obtained from TCGA database (http://cancergenome.nih.gov/).

### Patients and tissue samples

Fifty paired HCC primary and adjacent normal samples were obtained from patients who underwent surgrical treatment at the Xijing Hospital of the Fourth Military Medical University (Xi'an, China), which was under the supervision of the Hospital's Protection of Human Subjects Committee. None of our study patients had received chemotherapy or radiotherapy before the surgery, and all tissues were paraffin‐embedded after RNA and protein isolation. Written informed consent was obtained from all patients who donated tissue samples.

### RNA isolation and real‐time PCR analysis

miRNA was isolated using a Qiagen miRNeasy kit (Qiagen, Duesseldorf, Germany) according to the manufacturer's protocol. Then, reverse transcription of RNA to cDNA was performed (TaKaRa, Kumanoto, Japan). The expression levels of the miRNA and internal control (U6) were measured using quantitative real‐time PCR (Bio‐Rad, Hercules, CA, USA). The primer sequence of miR‐204‐5p was as follows: 5′TTCCCTTTGTCATCCTATGCCT3′. U6 and miRNA common primers were also purchased from TaKaRa. miRNA values were normalized to U6 expression before comparison.

### Cell culture and treatment

The human HCC cell lines BEL7402 and BEL7404 were purchased from the Cell Bank of the Chinese Academy of Sciences. Cells were incubated at 37 °C in a humidified atmosphere containing 5% CO_2_ and cultured in DMEM (Gibco, ThermoFisher Scientific, Inc., Grand Island, NY, USA) combined with 10% fetal bovine serum (FBS). miR‐204‐5p mimics and inhibitors were purchased from QIAGEN. For transient transfection of cells in six‐well plates, 100 μm mimics or inhibitors was added with HiPerFect reagent (QIAGEN) in OPTI‑MEM media (Gibco; ThermoFisher Scientific, Inc.) according to the manufacturer's instructions. The cells were plated in 10‐mm^2^ dishes and transfected with miR‐204‐5p overexpression and inhibition lentivirus (GeneChem, Shanghai, China). The inhibition lentivirus we used resulted in competitive inhibition. The stable cell lines were cultured with DMEM containing 2 μg·mL^−1^ puromycin (Merck Millipore, Darmstadt, Germany). The *SIX1* siRNA were purchased from GenePharm (Shanghai, China), and sequences are as follows: si1, 5′‐AGAAUAGUUUGAGCUCCUG‐3′ si2, 5′‐CACGCCAGGAGCTCAAACT‐3′ and Si3, 5′‐CCAGCTCAGAAGAGGAATT‐3′.

### CCK8 assay and BrdU test

For Cell Counting Kit‐8 (CCK8; Dojindo, Kumanoto, Japan) assays, cells were plated into 96‐well plates at a concentration of 3000 cells per well after transfection with different lentiviruses. According to the manufacturer's instructions, the cells were incubated with CCK8 for 4 h, and cell viability was evaluated by measuring the absorbance at 490 nm in a microplate absorbance reader (ThermoFisher Scientific). The assay was performed every 24 h for 5 days. For BrdU tests, cells were plated into 96‐well plates at 2 × 10^4^ cells with 100 μL DMEM. The next day, BrdU detection antibody (Merck Millipore) was added into the well according to the manufacturer's instructions. The cell viability was evaluated by the absorbance at a dual wavelength of 450/550 nm in a microplate absorbance reader.

### Colony‐forming assay

Cells were seeded into six‐well plate (1000–1500 per well) for 2 weeks. Then, the cells were fixed with absolute ethyl alcohol and dyed with 5% crystal violet. The positive cells turned blue and were counted.

### Subcutaneous xenograft mouse model

The animal experiments were approved by the Animal Ethics Committee of the Fourth Military Medical University (Xi'an, China). BALB/c‐nu/nu mice were purchased from the Experimental Animal Centre of the Fourth Military Medical University (Xi'an, China). BEL7404 cells transfected with miR‐204‐5p mimics, inhibitors and negative control were injected into the subcutaneous tissue at the dose of 2 × 10^6^ mL^−1^ (*n* = 5 per group). The tumours were separated and weighted when animals were sacrificed after 4 weeks. The tumour volumes were calculated once a week by the formula: *V* = (length × width^2^)/2 31.

### Dual‐luciferase reporter assay

The effects of miR‐204‐5p overexpression and knockdown on the SIX1 activity were measured by dual‐luciferase reporter assays. The BEL7402 cells were transfected with the wild‐type (WT) or MUT 3′ UTR of *SIX1*, which was inserted into the pGL3 vector in 96‐well plates. After 24 h, the dual‐luciferase assays were performed using the dual‐luciferase reporter assay system (Promega, Madison, WI,USA) with a Victor X machine (PerkinElmer, Norwalk, CT, USA).

### Western blotting

The whole cell protein was extracted using RIPA buffer combined with protease inhibitor (Merck Millipore). The protein concentration was determined by the Bradford method (Beyotime, Shanghai, China), and 20 μg of protein was separated by 10% SDS/PAGE (Beyotime) and transferred to polyvinylidene fluoride membrane (Merck Millipore). Then, the primary antibodies were incubated at 4 °C overnight and secondary antibodies for 1 h at room temperature. The primary antibodies used were mouse anti‐β‐actin (Sigma, USA), rabbit anti‐SIX1 (Proteintech, Rosemont, IL, USA), PCNA, c‐myc, cyclin‐D1 and cyclin‐A1 (Cell Signaling Technology, USA).

### Flow cytometry

Cell synchronization was obtained using a nutritional deprivation strategy. Cells transfected with microRNA mimics, inhibitors and negative controls were incubated with serum‐free DMEM (Gibco) for 24 h for cell synchronization. Then, the cells were treated with 10% FBS‐containing DMEM, trypsinized and washed at 0, 1, 3, 6, 9 and 12 h postrelease from FBS‐free DMEM. The cells were fixed in 70% ethanol at 20 °C overnight. For flow cytometry assays, the fixed cells were stained with propidium iodide solution (Sigma) and incubated at 37 °C in the dark for 30 min. DNA content was examined by flow cytometry using a FACSCalibur.

### Diethylnitrosamine(DEN)‐induced mouse HCC model

The DEN‐induced mouse HCC model was generated as described previously 32. Briefly, 12‐ to 15‐day‐old male mice were injected with DEN, which was diluted in phosphate‐buffered saline (PBS, pH 7.4; Gibco) to a final DEN concentration of 5 mg·mL^−1^. The DEN was intraperitoneally injected at 25 μg per gram of body weight. After 10 months, the mice were sacrificed for tumour protein and miRNA extraction.

### Construction of tissue microarray (TMA)

The construction of the TMA was performed as described previously 33. One slide of the TMA was H&E‐stained for histological verification, and samples where the tumour tissue occupied > 10% of the core area were thought to be qualified.

### Immunohistochemical staining

Hepatocellular carcinoma TMAs (Outdo Biotech, Shanghai, China) were deparaffinized with dimethylbenzene and alcohol of different concentrations (100%, 95%, 85%, 75%) in sequence. Then, the TMA was boiled in a high‐pressure kettle with citrate buffer for antigen retrieval. Endogenous peroxidize was blocked using 0.5% H_2_O_2_ for 20 min, and nonspecific binding was inhibited by 10% goat serum for 30 min at room temperature. After removing the goat serum, the TMA was incubated with primary antibody (1 : 250; Proteintech) at 4 °C overnight. Then, the biotinylated anti‐mouse secondary antibody was added for 1 h and streptavidin–HRP for 30 min. Next, the TMA was stained DAB (DAB chromogen/DAB substrate = 1 : 20) and counterstained with haematoxylin and eosin (H&E). Finally, the TMA was washed and dehydrated using the reverse order of the gradient alcohol and dimethylbenzene, which were used in the dewaxed protocol. The primary antibodies used were mouse anti‐β‐actin (Sigma), rabbit anti‐SIX1 (Proteintech), PCNA, c‐myc, cyclin‐D1 and cyclin‐A1 (Cell Signaling Technology).

### Evaluation of immunostaining intensity

Semiquantitative scoring of intensity was conducted by two independent pathologists. The scoring system consisted of two different parameters, the percentage of positive cells and the intensity of staining. The percentage of cells was classed into four levels, representing different percentage scores: < 1% (0), 1–25% (1), 26–75% (2) and > 75% (3). The intensity of staining was also classed into four levels: negative (0), weak (1), moderate (2) and strong (3). The final score equals the product of the two parameters and was graded as low (final score 0–3) and high (final score 4–9), which represented ‘SIX1 low expression’ and ‘SIX1 high expression’.

### Statistical analysis

The statistical analysis was performed using spss 22.0 software (Chicago, IL, USA). Data are presented as the mean ± SEM and were compared between two groups by Student's unpaired *t*‐test. The comparisons between the control group and several experimental groups were made by one‐way ANOVA. Continuous data were compared by two‐way ANOVA. To assess the expression of miR‐204‐5p between HCC tissues and corresponding adjacent normal tissues, the paired *t*‐test was used. The relationship between miR‐204‐5p and SIX1 was calculated using chi‐square test. The disease‐free survival of patients and comparisons were plotted using the Kaplan–Meier method and univariate log‐rank test. Univariate and multivariate analysis, hazard ratios (HRs) and their 95% confidence intervals (CIs) were assessed using the Cox regression model. *P* < 0.05 was considered statistically significant, and all *P* values were two‐sided.

## Author contributions

JL designed the study and edited the manuscript. YC, MZJ, FD, DC, TY, ZYQ, BX, XWL, WJW and HML performed the experiments. YC and MZJ analysed the data and wrote the manuscript. YZN critically revised the manuscript. DMF supervised the project and advised with regard to the experimental design.

## Supporting information


**Fig. S1.** miR‐204‐5p suppressed Huh7 cells proliferation *in vitro*.
