# Supplementary figures and images for: miR‐204‐5p suppresses hepatocellular cancer proliferation by regulating homeoprotein SIX1 expression
Source: FEBS Open Bio. 2018 Jan 15;8(2):189–200. doi: 10.1002/2211-5463.12363 (PMC5794460; doi:10.1002/2211-5463.12363)

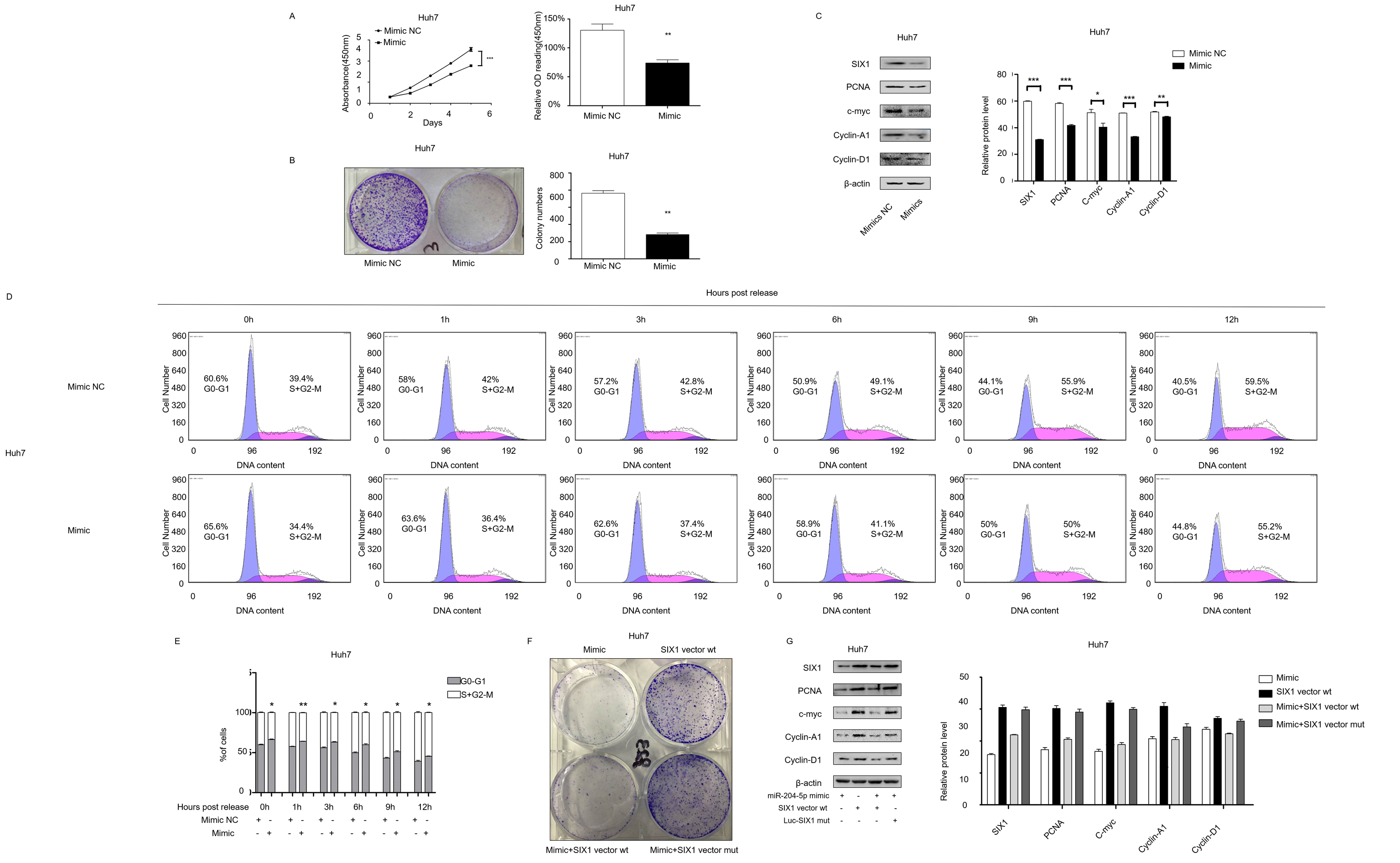

Supplement: Supplementary file 1 — Fig. S1. miR‐204‐5p suppressed Huh7 cells proliferation in vitro. [file FEB4-8-189-s001.jpg]
